# Systems analysis of human brain gene expression: mechanisms for HIV-associated neurocognitive impairment and common pathways with Alzheimer’s disease

**DOI:** 10.1186/1755-8794-6-4

**Published:** 2013-02-13

**Authors:** Andrew J Levine, Jeremy A Miller, Paul Shapshak, Benjamin Gelman, Elyse J Singer, Charles H Hinkin, Deborah Commins, Susan Morgello, Igor Grant, Steve Horvath

**Affiliations:** 1 Department of Neurology, National Neurological AIDS Bank, David Geffen School of Medicine at the University of California, Los Angeles, CA, USA; 2 Department of Human Genetics, David Geffen School of Medicine at the University of California, Los Angeles, CA, USA; 3 Department of Medicine (Division of Infectious Disease & International Medicine) and Department of Psychiatry & Behavioral Medicine, Morsani College of Medicine, University of South Florida, Tampa, FL, USA; 4 Departments of Pathology and Neuroscience & Cell Biology, University of Texas Medical Branch, Galveston, USA; 5 Department of Psychiatry and Biobehavioral Sciences, David Geffen School of Medicine at the University of California, Los Angeles, CA, USA; 6 VA Greater Los Angeles Healthcare System, Los Angeles, CA, USA; 7 Department of Pathology, Keck School of Medicine at the University of Southern California, Los Angeles, CA, USA; 8 Departments of Neurology, Neuroscience, and Pathology, Manhattan HIV Brain Bank, The Mount Sinai School of Medicine, New York, USA; 9 Department of Psychiatry, California NeuroAIDS Tissue Network, University of California, San Diego, USA; 10 Department of Biostatistics, University of California, Los Angeles, CA, USA

**Keywords:** HIV encephalitis, HIV-associated dementia, HIV-associated neurocognitive disorder, Weighted gene coexpression network analysis, WGCNA, CNS penetration effectiveness, National neuroAIDS tissue consortium, Coexpression module

## Abstract

**Background:**

Human Immunodeficiency Virus-1 (HIV) infection frequently results in neurocognitive impairment. While the cause remains unclear, recent gene expression studies have identified genes whose transcription is dysregulated in individuals with HIV-association neurocognitive disorder (HAND). However, the methods for interpretation of such data have lagged behind the technical advances allowing the decoding genetic material. Here, we employ systems biology methods novel to the field of NeuroAIDS to further interrogate extant transcriptome data derived from brains of HIV + patients in order to further elucidate the neuropathogenesis of HAND. Additionally, we compare these data to those derived from brains of individuals with Alzheimer’s disease (AD) in order to identify common pathways of neuropathogenesis.

**Methods:**

In Study 1, using data from three brain regions in 6 HIV-seronegative and 15 HIV + cases, we first employed weighted gene co-expression network analysis (WGCNA) to further explore transcriptome networks specific to HAND with HIV-encephalitis (HIVE) and HAND without HIVE. We then used a symptomatic approach, employing standard expression analysis and WGCNA to identify networks associated with neurocognitive impairment (NCI), regardless of HIVE or HAND diagnosis. Finally, we examined the association between the CNS penetration effectiveness (CPE) of antiretroviral regimens and brain transcriptome. In Study 2, we identified common gene networks associated with NCI in both HIV and AD by correlating gene expression with pre-mortem neurocognitive functioning.

**Results:**

Study 1: WGCNA largely corroborated findings from standard differential gene expression analyses, but also identified possible meta-networks composed of multiple gene ontology categories and oligodendrocyte dysfunction. Differential expression analysis identified hub genes highly correlated with NCI, including genes implicated in gliosis, inflammation, and dopaminergic tone. Enrichment analysis identified gene ontology categories that varied across the three brain regions, the most notable being downregulation of genes involved in mitochondrial functioning. Finally, WGCNA identified dysregulated networks associated with NCI, including oligodendrocyte and mitochondrial functioning. Study 2: Common gene networks dysregulated in relation to NCI in AD and HIV included mitochondrial genes, whereas upregulation of various cancer-related genes was found.

**Conclusions:**

While under-powered, this study identified possible biologically-relevant networks correlated with NCI in HIV, and common networks shared with AD, opening new avenues for inquiry in the investigation of HAND neuropathogenesis. These results suggest that further interrogation of existing transcriptome data using systems biology methods can yield important information.

## Background

Infection with Human Immunodeficiency Virus-1 (HIV) frequently leads to neurologic consequences that are heterogeneous both behaviorally and pathologically. Behaviorally, some individuals will develop HIV-associated neurocognitive disorder (HAND), a syndrome of widely varying severity with unclear neuropathogenesis [1]. In some individuals with HAND, pathological evidence of encephalitis, termed HIV-encephalitis (HIVE), is seen upon autopsy; however, the correlation between HAND and HIVE is modest at best [2,3]. To investigate the neuropathogenesis of HIVE and HAND, several methodologies have been employed, including measurements of gene expression alterations using. To date, most transcriptome studies utilizing brains of HIV-infected (HIV+) humans have focused on gene expression changes underlying HIVE. Such studies, generally limited to examination of the frontal grey matter, have found altered regulation of genes involved in neuroimmune functioning, and also implicated neurodegenerative pathways based on dysregulation of genes involved in synapto-dendritic functioning and integrity [4], toll-like receptors [5], and interferon response [6]. As recently reviewed [7], findings from human microarray studies have been partially replicated in simian immunodeficiency virus models, especially with regards to interferon-related and neuroinflammatory-related genes [8,9]. Mouse astrocytes exposed to HIV have also shown some transcription overlap with those of simian and human studies [10]. More recently, in an analysis of gene microarray data derived from multiple brain regions of HIV + individuals with HAND alone or HAND with HIVE, two apparently distinct transcriptome profiles were found, suggesting that there may be two etiological pathways to HAND [11]. Specifically, HIVE with concomitant HAND was associated with high brain viral load, upregulation of inflammatory pathways across all brain regions, and downregulation of neuronal transcripts in frontal neocortex. Conversely, HAND without HIVE was characterized by low brain HIV burden without evidence of increased inflammatory response, and without downregulation of transcripts in frontal neocortical neurons. Indeed, only transcripts characteristically expressed by vascular and perivascular-type cells were consistently dysregulated in HAND without HIVE. Taken together, the results of that most recent and comprehensive study of HIV+ brain transcriptome suggests divergent neuropathogenic gene expression signatures reflecting different pathways underlying HAND with HIVE as compared to HAND without HIVE (see also Gelman and Moore [12]).

While these studies have provided important information, further interrogation of the data may provide further insight into the pathogenesis of HAND and HIVE. Thus far, analysis of transcriptome data has involved comparing gene transcript levels between groups and then determining the gene ontology of those genes that demonstrate significant differences in expression. However, because genes function in biologically-related networks, and therefore are likely to exhibit correlated expression, this method may overlook important aspects of disease pathogenesis. Further, while similarities may be observed in the expression of specific genes across species, these genes may be involved in different biological pathways in the species examined, confounding interpretation. As such, despite the diversity of microarray studies of HIV+ brains within and across species, no coherent picture of transcriptome aberrations has emerged. In the current study, we apply weighted gene co-expression network analysis (WGCNA), a proven systems biology methodology, to an existing gene expression data set derived from HIV + human brains [13-15]. WGCNA enables a more systematic and global interpretation of gene expression data, identifying biologically meaningful ‘modules’ that are often comprised of functionally related genes and/or correspond to cell types [16]. This is accomplished by assessing gene co-expression patterns (i.e., correlation matrices). The focus on co-expression modules, each consisting of possibly hundreds of genes with common co-expression across samples, allows for a biologically motivated reduction of data while also alleviating the problem of multiple comparisons. Typically, WGCNA results in fewer than 20 modules (as opposed to thousands of genes), which can then be examined for their association to clinical or biological variables of interest. Given the limited sample size of most transcriptome studies, a module based analysis is not only biologically meaningful but also attractive from a statistical point of view. An advantage of WGCNA *vis*-*a*-*vis* alternative coexpression based analyses is the availability of powerful module preservation statistics which can be used to quantitate the extent to which disease related modules are present in other data sets, including other species, or other organs and cells within the same organism [17-19]. Finally, by reducing data into a few biologically meaningful co-expression modules, WGCNA allows for direct association analysis with clinical and biomarker variables, thus allowing direct delineation of causative disease pathways. The validity of WGCNA is demonstrated by the reproducible and biologically meaningful results in several other applications and diseases [20-24].

Another highly useful application of microarray data is to identify disease mechanisms that are common between neurologic conditions. Studies of transcriptomic changes have been vital in elucidating the pathogenesis of neurodegenerative conditions such as Alzheimer’s disease (AD ) [25-27]. Utilizing such data, Miller et al. employed WGCNA to explore commonalities and differences between normal aging and pathological aging (i.e. AD) [28], resulting in the identification of biologically relevant modules conserved between AD and aging that included mitochondrial processes and synaptic plasticity. In the context of HAND, identifying biological pathways common to Alzheimer’s disease and other neurological conditions could open the door to employing readily available pharmaceutical treatments in those with HIV.

In this paper we describe two studies. In Study 1, we extended the findings of Gelman et al. [11] in a number of ways. First, we examined the relationship between gene expression and two clinically-relevant variables: HIV-related neurocognitive impairment (regardless of HIVE status) and CNS penetration effectiveness [29] of antiretroviral drug regimens. We focused on neurocognitive impairment as this is a more continuous phenotype, as compared to neuropathological diagnosis. We also chose to examine CPE as the effect of highly penetrant regimens upon neurobiological functioning remains uncertain. We utilized both standard differential expression and WGCNA to identify biologically relevant pathways and modules associated with these clinical variables. We also utilized WGCNA to identify co-expression module differences across four clinically and/or neuropathologically distinct groups: HIV-seronegative, HIV + without HAND or HIVE, HAND without HIVE, and HAND and HIVE. We hypothesized that WGCNA would allow further and valuable interpretation of previously evaluated microarray data, and that biologically-relevant pathways and modules associated with clinical variables would be identified. In Study 2, we elucidated transcriptional commonalities and differences in neurocognitive impairment among HIV and AD. We hypothesized that both diseases would involve common genes and pathways associated with neurocognitive impairment.

## Methods

### Study 1: Identifying transcriptome networks associated with neurocognitive impairment and CNS penetration effectiveness of antiretroviral regimen using both standard differential gene expression analysis and WGCNA

#### Subject information/data acquisition

Data were obtained from the National NeuroAIDS Tissue Consortium [30], as described previously [11]. The National NeuroAIDS Tissue Consortium consists of four study sites, in New York City (PI – Susan Morgello, M.D.), Los Angeles (PI – Elyse Singer, M.D.), San Diego (PI – Igor Grant, M.D.), and Galveston (PI – Benjamin Gelman, M.D., Ph.D.). Studies were conducted in accordance with human subject protection protocols as approved by the Institutional Review Boards at the respective sites. Written consent was obtained for subjects. Subject assessment procedures, and specimen and RNA processing were previously described [11]. In the original study, there were 24 cases divided into 4 groups according to criteria described below: Group A (N = 6): HIV uninfected with no neuropathological abnormalities at autopsy; Group B (N = 6): HIV + neurocognitively normal with no neuropathology; Group C (N = 7) HIV + with severe HAND (defined below) with no HIVE or substantial neuropathological defect; Group D (N = 5) HIV + with HAND and HIVE. All HIV cases were evaluated within six months of death. HIV status was determined via enzyme-linked immunosorbent or polymerase chain reaction assay using blood collected pre-mortem. HIVE was diagnosed via post-mortem neuropathological examination. HAND status was determined via the Global Clinical Rating (GCR). The GCR is a summary score based on an individual’s performance across a battery of neurocognitive tests. A GCR score of 1–4 indicated performance ranging from above average to normal; GCR of 5–9 indicated progressively severe impairment.

For the current study, 21 of the 24 cases were used: two cases from group B were removed due to poor quality RNA and one case from group D was removed due to presence of brain-related opportunistic infection upon neuropathological examination. Specifically, sample WM-A6-06, and all three samples from subjects B5, B6, and D5 were removed. RNA was processed using the Affymetrix® gene array platform (human 133 plus 2.0). Three brain regions were examined: frontal neocortex (FC), neostriatum (BG), and frontal white matter (WM). Table 1 displays pre-mortem participant characteristics and post-mortem interval (PMI: time between death and removal of brain). In Study 1, the grouping method employed by Gelman et al. [11] was used only for the initial WGCNA to determine if this interrogative method was of added value. For purposes of this study, those with scores above 6 were considered to have HAND. This level of neurocognitive impairment is consistent with severe HAND, or HIV-associated dementia [1].

**Table 1:** **Group characteristics from Gelman et al., 2012**[70]

**Group (N)**	**HIV status**	**Neurocognitive Impairment (GCR)**	**HIVE**	CPE*	**Age (years)**	**PMI (hours)**
A (6)	Negative	No (NA)	NA	NA	50 ± 10	19 ± 5.1
B (4)	Positive	No (2.8 ± 1.3)	No	2.1 ± 0.9	50 ± 6.5	11 ± 8.1
C (7)	Positive	Yes (7.1 ± 0.7)	No	1.3 ± 2	44 ± 9.8	7.2 ± 2.1
D (4)	Positive	Yes (8.3 ± 1.7)	Yes	1.8 ± 0.3	44 ± 10	11 ± 4.8

* Note that this grouping schema was used only on one analysis in Study1.

** CNS Penetration Effectiveness. Higher values indicate greater penetration of antiretroviral medications into brain.

Study 1 also included correlation of gene expression with the clinical variables described next. These data were obtained within six months prior to death.

##### CNS penetration effectiveness (CPE)

The reported effects of antiretroviral medications upon neurocognitive functioning ranges from beneficial [31,32] to detrimental [33]. As such, we sought to determine the impact of antiretroviral regimen on gene expression. We employed the CPE, a score that is based on the pharmacokinetic and pharmacodynamic characteristics of antiretroviral medications [29]. CPE scores for the last known regimen were calculated. Higher scores indicate a regimen with increased penetration of the blood brain barrier and therefore putatively greater impact on neurobiological activity.

##### Neurocognitive impairment (NCI)

NCI is the key feature of HAND and is often association with post-mortem HIVE. We sought to determine transcriptional correlates of neurocognitive functioning, as reflected by the GCR (described above) in HIV + cases only. Used as a continuous variable in the current paper, higher scores indicate greater impairment.

#### Statistical analyses

##### Data preprocessing

Gene array data was received in the form of Affymetrix .cel files. These files were then read into R and preprocessed using the *espresso* function and the MAS5 method of preprocessing. We chose MAS5 based on a study by Lim et al. [34], which found that MAS5 provides the most faithful cellular network reconstruction of the four commonly used normalization procedures (MAS5, RMA, GCRMA and Li-Wong). Our unbiased array outlier detection method considered metrics measuring RNA quality and low inter-array correlations. First, we calculated the un-normalized inter-array correlation using the Pearson correlation [35]. Hierarchical clustering based on the inter-array correlations revealed seven outlying arrays (all samples from two subjects in group B, and the WM sample from one subject in group A). The majority of these samples also were found to have poor quality RNA during the quality control step, suggesting that this outlier removal method based on hierarchical clustering is reasonable. We also removed all samples from one individual in group D due to the presence of confounding CNS co-infections (Cytomegalovirus and Papovavirus). Quantile normalization was then performed on the remaining data. Probe sets that were called “absent” in >90% of the samples by MAS5 preprocessing were excluded from further consideration. In order to decrease computational constraints and facilitate between-study comparisons, the function *collapseRows* in the WGCNA library was used to select a single probe set for each gene. Our studies show that the most reproducible representative probe set is the one with the highest average expression across samples [36]. Probe sets without associated gene symbols were omitted. In summary, each gene symbol was represented by a single probe set.

#### Differential expression analyses

The relationship between each gene and the two clinical variables (NCI and CPE) were assessed across three brain regions (FC, WM, and BG) using Pearson correlation. The Pearson correlation test is appropriate since it only assumes that one of the quantitative variables (here gene expression) follows a normal distribution. Results of these differential expression analyses were used in three ways. First, enrichment analyses were performed on all differentially expressed genes (p < 0.05 in all cases) using Expression Analysis Systematic Explorer (EASE) [37]. EASE functionally categorizes gene lists based on enrichment for all levels of the gene ontology (GO), Kyoto Encyclopedia of Genes and Genomes (KEGG), or SwissProt terms, and returns an EASE score (similar to a p-value) for assessing significance. Second, the trait-gene relationship values (i.e., correlations or T-values) for all genes were retained and converted into a color vectors for visual assessment of disease-gene relationships in the network analysis. In addition to NCI and CPE, color vectors for HIV and HIVE status were produced. Finally, to further explore network functioning of genes implicated in NCI and CPE, genes with the highest correlations were entered into GNCPro (http://GNCPro.sabiosciences.com) [38] to understand additional biological relationships. GNCPro, free online software developed and maintained by SABiosciences, is an *in silico* research tool for collating gene and pathway interactions. GNCPro integrates collective biological knowledge through text mining, data mining, data acquisition and computational prediction. The interactions among a group of genes are represented graphically and are interactive. Gene co-expression relationships are designated as edges and the genes and proteins are designated as nodes. Gene co-expression relationships are calculated as previously described [39] and Shannon’s Entropy formula is used to calculate human gene tissue expression profiles [40]. Gene groups are identified by categories including expression, interaction, modification, and regulation (http://GNCPro.sabiosciences.com). In addition, it should be noted that the GNCPro program has an upper limit of 100 genes that can be shown in any network analysis, including the input genes.

#### Weighted gene co-expression network analysis

Here we employ for the first time WGCNA to the analysis of HIV brain transcriptome data. The WGCNA started out from 14099 genes assayed, identifies modules of co-expressed genes, and relates these modules to clinical variables and gene ontology information [13,15,41]. Because gene modules may correspond to biological pathways, focusing the analysis on modules (and their highly connected intramodular hub genes) amounts to a biologically meaningful data reduction scheme. Highly correlated module genes are represented and summarized by their first principal component (referred to as the module eigengene, or ME [42]). The ME is used to define measures of module membership which quantify how close a gene is to a given module. Module membership measures allow one to annotate all genes on the array and to screen for disease related intramodular hub genes [15,43,44]. As described below, we use functional enrichment analysis with regard to known gene ontologies to understand the biological significance of the ME and to identify putative disease pathways.

WGCNA was performed separately for each of the three brain regions using the most highly expressed probe for each gene present in at least six samples, as previously described [15,35,41]. In short, for each analysis the Pearson correlations between all genes across all relevant samples were calculated. We calculated a signed-weighted co-expression adjacency matrix as follows:

$$\text{adjacency}={\left[\left(\text{correlation}+1\right)/2\right]}^{12}$$

This allowed us to keep track of the direction of the correlation. Note that a correlation of −1 and +1 lead to an adjacency value of 0 and 1, respectively. Recent studies have shown that signed co-expression networks can be superior when it comes to detecting small modules [21]. The power 12 is the default soft threshold parameter for constructing a signed weighted network [21,41]. Topological overlap (TO), a more biologically meaningful measure of node interconnectedness (similarity), was then calculated as described previously [35,41,45,46]. Next, genes were hierarchically clustered using 1 − TO as the distance measure and modules were determined by using a dynamic tree-cutting algorithm [47].

To enhance reader friendliness the module labels of different data sets were relabeled using the WGCNA function *matchLabels*. This function compares the genes comprising each module in both data sets using a hypergeometric test, then relabels modules in the second data set so that modules with a significant number of overlapping genes between data sets will have the same label. For example, if the Blue module in the second data set corresponds to the Turquoise module in the first data set, then we relabeled the Blue module as Turquoise module. Thus, modules with the same label have a significant number of overlapping genes, but not identical genes, and modules with labels unique to a single data set were not recapitulated in the other data sets.

The expression profiles of genes inside a co-expression module were summarized by the ME, which is the first principal component. Note that the ME is the mathematically optimal summary of the module expression profiles since it captures the maximum amount of variation [42,48]. In the current study, the MEs were used in two analyses. First, they were compared across groups A, B, C, and D using ANOVA. Second, they were correlated to both of the clinical variables (NCI and CPE) to identify trait related modules. To further enhance data interpretation, we used EASE to characterize modules (as described above), and also used the function *userListEnrichment* from the WGCNA library to find enrichment for cell type markers and other brain-related categories. Additional details and related software tutorials can be found at our webpage: http://www.genetics.ucla.edu/labs/horvath/CoexpressionNetwork/.

### Study 2: differential gene expression and enrichment analysis to determine common pathways associated with neurocognitive impairment in HIV and Alzheimer’s disease

#### Patient groups

All HIV + cases (Groups B, C, and D, as described above) were used. Data for Alzheimer’s (AD) cases was produced in a previous study (Blalock et al., 2004) [25] and available on the Gene Expression Omnibus. In that study, AD cases were clinically evaluated and their diagnosis was confirmed at autopsy. After removing 3 outliers (see [28]), there were 8 controls, 6 cases with incipient (mild) dementia, 8 with moderate dementia, and 6 with severe dementia. Dementia severity was determined primarily via pre-mortem neurocognitive testing. Gene expression data for both studies were profiled using Affymetrix® gene array platforms (HG-U133A).

#### Primary clinical outcomes

Study 2 included correlation of gene expression with neurocognitive impairment. As in Study 1, neurocognitive impairment was determined using the GCR in HIV + cases, as described above. In AD cases, the Mini Mental Status Exam, or MMSE [49], was used. The MMSE is a widely used neurocognitive screening measure. Scores range from 0–30, with lower scores indicating greater impairment.

#### Statistical analysis

To assess the transcriptional similarities between NCI associated with HIV and AD, gene microarray data from FC and BG of the 15 HIV+ brains (Groups B, C, and D described above) were compared to that of hippocampus of 30 brains obtained from individuals from the Blalock et al. study, including 8 neurocognitively normal controls and 22 with autopsy-confirmed AD [25].

First, we chose a single probe for each gene in the AD analysis using *collapseRows* as with these data, omitting all genes except the ones common to both analyses (total common genes = 8167) [36]. Second, we calculated the correlation between transcription level of each of these genes and the MMSE score in the AD data for comparison with the correlations between the respective genes and GCR scores in the HIV + group. We then plotted each of these pairs of correlations as a point on a graph in order to assess to what extent impairment genes in HAND are also impairment genes in AD. We define "impairment genes" as genes whose expression either increases (upper right of plots; R > 0.25) or decreases (lower left of plots; R < −0.25) with impairment in the hippocampus of AD subjects, as well as both the BG and FC of HAND subjects. We then conducted enrichment analysis on significant genes. In addition, we assessed which of the modules in the WGCNA of HIV + brains were also enriched for AD-related impairment genes by comparing the number of genes in the Blalock study that were correlated with MMSE score with the number expected by chance using a hypergeometric test.

**Figure 1:**
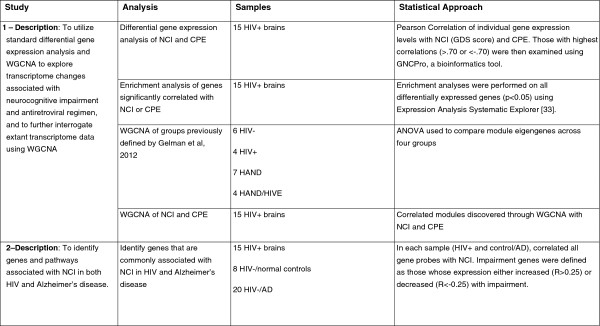
Overview of studies and analyses, in the order that they appear in the results.

An overview of both studies and associated analyses are depicted in Figure 1.

## Results

### Study 1: Standard differential gene expression analysis of clinical variables

The relationship between individual gene expression and both clinical variables, CPE and NCI, are described here. While no genes achieve significance after correcting for multiple comparisons, we still present the top genes as potential genes of interest for future studies, but note that many are expected to be false positives. The fact that these results are not significant after adjusting for multiple comparisons highlights a major pitfall of standard differential expression analysis. In contrast, module based analyses provided by WGCNA largely circumvent the multiple comparison problem as shown below.

#### CPE

All genes with an absolute correlation co-efficient (i.e., positive or negative) value of .75 or greater are listed in Table 2. Correlations are between absolute gene expression and CPE score. Additional correlations are provided in the Supplemental Data [Additional file 1 - Gene Correlations with NCI and CPE.xls].

**Table 2:** Top correlations between genes and CPE in each brain region

**Frontal cortex**			**Basal ganglia**			**White matter**		
**Gene name**	**R**	P-value*	**Gene name**	**R**	P-value*	**Gene name**	**R**	P-value*
WBSCR16	0.82	0.0003	LOC730496	0.82	0.0003	TMEM57	−0.81	0.0006
MYST1	0.82	0.0004	CA13	0.82	0.0003	NFIC	0.80	0.0006
RPS6KB2	0.82	0.0004	CLEC14A	0.81	0.0004	KIAA0515	0.79	0.0007
ZNF26	0.81	0.0004	C18orf17	0.78	0.0010	CIDEB	0.79	0.0007
DCAKD	−0.79	0.0008	OSBPL6	−0.77	0.0012	RDH11	−0.79	0.0008
LYPLA2	0.78	0.0009	MRAS	0.77	0.0012	HMHB1	−0.79	0.0009
NME3	0.78	0.0010	SSR3	0.77	0.0012	VPS13D	0.78	0.0011
SLC10A2	0.76	0.0015	FUT2	0.77	0.0012	TAF5L	0.77	0.0012
TRIM31	0.76	0.0015	EHHADH	0.76	0.0014	TNPO3	0.77	0.0014
			MOSPD1	−0.76	0.0015	FLJ45803	0.76	0.0017
			SIKE	0.76	0.0016	C6orf113	−0.76	0.0018
			TMEM67	−0.76	0.0016	C19orf43	0.75	0.0018
			LASS4	0.76	0.0017	SH3GLP3	0.75	0.0018
			AGPAT6	0.76	0.0017			
			CCDC100	0.76	0.0017			
			ACVR2B	0.75	0.0018			
			SREBF2	0.75	0.0018			

* p-values shown are not corrected for multiple comparisons.

#### NCI

Genes with absolute correlation coefficients of greater than .75 are listed in Table 3. Additional results of this analysis are provided in the Supplemental Data [Additional file 1 Gene Correlations with NCI and CPE.xls].

**Table 3:** Top correlations between genes and NCI in each brain region

**Frontal cortex**			**Basal ganglia**			**White matter**		
**Gene name**	**R**	P-value*	**Gene name**	**R**	P-value*	**Gene name**	**R**	P-value*
GINS1	−0.80	0.0004	TP53BP2	0.85	0.00006	GNAZ	0.84	0.00009
ACADM	0.77	0.0007	COQ10A	0.82	0.0002	NIPSNAP3A	−0.82	0.0002
ATAD3B	0.76	0.0009	GINS2	−0.81	0.0003	ID2	−0.82	0.0002
C8orf48	0.76	0.0011	PCDHB6	−0.79	0.0005	ADO	0.80	0.0003
EFTUD2	−0.76	0.0011	RHOJ	0.78	0.0006	CHADL	0.79	0.0005
CLUAP1	−0.75	0.0013	LOC550631	−0.78	0.0006	GRK6	0.78	0.0006
			CADM1	0.78	0.0007	FBXL13	−0.78	0.0006
			FECH	−0.76	0.001	ARHGAP22	0.77	0.0007
			SFRS1	0.75	0.0011	CHST12	0.77	0.0008
						DAP	−0.76	0.0009
						PASK	−0.76	0.0010
						C9orf142	0.76	0.0011

* p-values shown are not corrected for multiple comparisons.

#### Detailed enrichment analysis of top genes related to clinical variables

To further understand gene expression pathways related to HIV-related brain dysfunction, we examined enriched GO categories associated with CPE and NCI in all three brain region based on genes identified in the differential expression analysis. After correcting for multiple comparisons, no categories were found to be associated with CPE. The results of the NCI analysis are shown in Table 4. After correcting for multiple comparisons, gene pathways involved in mitochondrial functioning, mitotic cell cycle, DNA repair, proteasome complex, and nucleus were downregulated in the FC. No significant downregulated pathways were found in the BG or WM. Upregulated pathways were more widely distributed across the brain, with some overlap between regions. For example, transcription regulation was upregulated in both the FC and WM, whereas the genes on Homo Sapiens chromosome 19 were enriched in the BG and WM. Other pathways, shown in Table 4, were significant in single regions.

**Table 4:** Enriched GO categories for genes related to neurocognitive impairment

**Down with impairment**	**Frontal cortex**	**Basal ganglia**	**White matter**
Mitochondrion	6.86E-6	NS	NS
Mitotic Cell Cycle	1.63E-04	NS	NS
DNA Repair	4.86E-04	NS	NS
Proteasome Complex (sensu Eukarya)	5.06E-04	NS	NS
Nucleus	1.10E-03	NS	NS
**Up with Impairment**	**Frontal Cortex**	**Basal Ganglia**	**White Matter**
Transcription Regulation	5.49E-06	NS	2.10E-03
Receptor Activity	3.39E-04	NS	NS
DNA Binding	1.34E-03	NS	NS
Protein Kinase Activity	NS	7.13E-04	NS
Homo Sapiens 19	NS	1.18E-02	2.27E-03
Chondroitin/Heparin Sulfate Biosynthesis	NS	NS	8.37E-05

**Figure 2:**
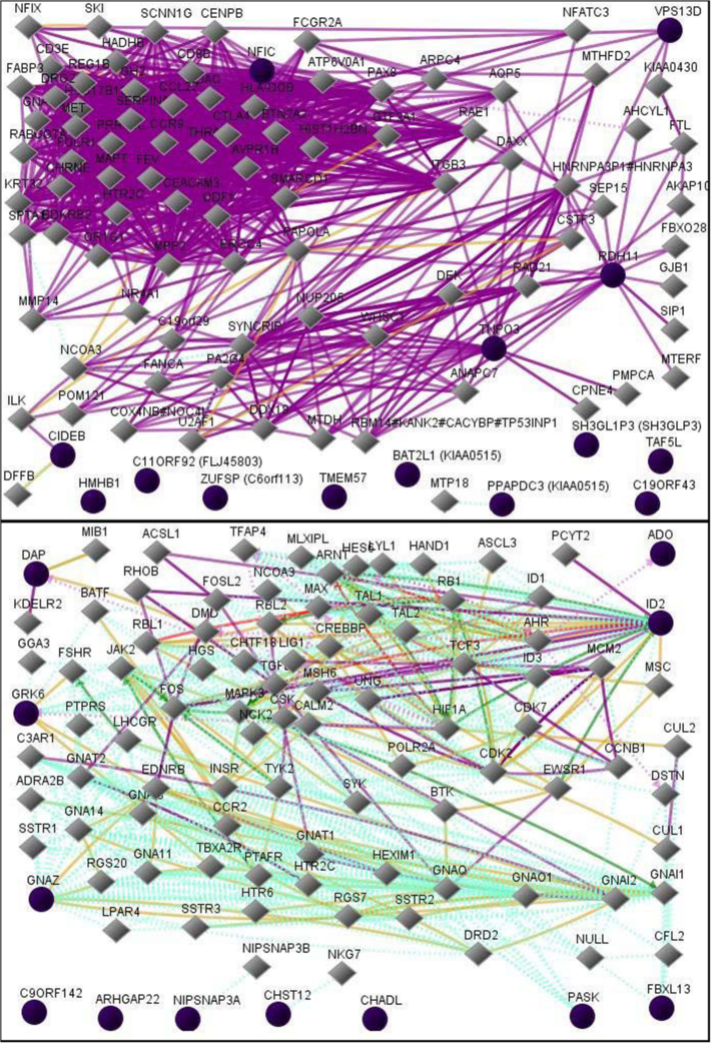
**Network diagram of CPE-related genes (top panel) and NCI-related genes (bottom panel) in white matter.** Input genes from Table 2 are indicated by solid black circles. The diamond-shapes are neighboring genes that are identified by literature-based associations. There are various types of interactions among the genes represented as lines and dotted lines in color as follows: Down-regulation – red; Up-regulation – green; Regulation – grey; Co-expression – purple; Physical Interaction – brown; Predicted Protein Interaction – dotted aquamarine; Predicted Transcription Factor Regulation – dotted brown.

To further interrogate these data, and since we were unable to find significant enrichments for CPE using traditional GO enrichment analysis, we expanded our results into the known literature using GNCPro [38], as described above. Genes listed in Tables 2 &3 were entered into GNCPro allowing visualization of various types of interactions among the identified genes. We include only diagrams of the WM for both CPE and NCI (Figure 2). We chose WM based on the current available literature, which indicates that antiretroviral regimen has the most conspicuous metabolic effects within this region [50]. (Diagrams for remaining regions are available as Supplemental material). For CPE, co-expression connectivity was the most prevalent of the several types of connectivity identifiable by GNCpro. We observe high connectivity of transportin 3 (TNPO3). TNPO3 is necessary for full integration of HIV-1 into the host DNA [51,52]. Counter intuitively, expression of this gene was positively correlated with CPE in the WM. Also observed to have high co-expression connectivity in the WM, although of less clear relation to CPE, were retinol dehydrogenase 11 (RDH11), vacuolar protein sorting 13 (VPS13D), and nuclear factor I/C (NFIC). For NCI, the most common types of connectivity indicated through GNCPro were protein-protein interactions and physical interaction. However, for one gene, Inhibitor of DNA Binding (ID2), there was a high diversity of interactions. Another apparently relevant gene showing modest connectivity is death-associated protein (DAP). Both ID2 and DAP were negatively correlated with NCI. A third gene, G protein-coupled receptor kinase 6 (GRK6), was positively correlated with NCI. A final highly connected gene within this network, of unclear importance, was guanine nucleotide-binding protein – alpha Z polypeptide (GNAZ).

#### Weighted gene co-expression network analysis

**Figure 3:**
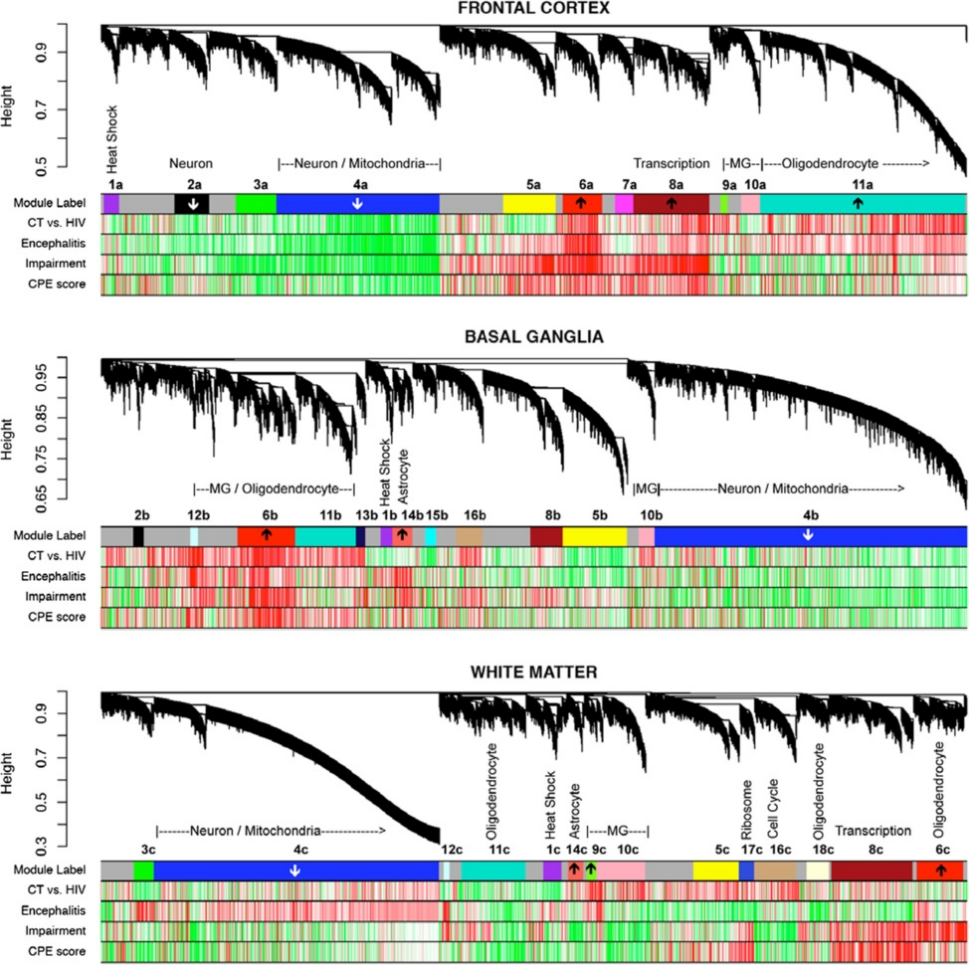
**Co-expressed genes tend to cluster into modules corresponding to major biological divisions (cell type, molecular function, etc.), as well as disease-relevant changes.** Module summaries are presented for frontal cortex (top), neostriatum (middle), and white matter (bottom). The dendrograms group genes into distinct modules using hierarchical clustering. The y-axis corresponds to distance determined by the extent of topological overlap (1-TO). Top color band (Module label): dynamic tree cutting was used to identify highly parsimonious module definitions, generally dividing modules at significant branch points in the dendrogram. Second color band (CT vs. HIV): plotted T values of control vs. HIV + samples. Third color band (Encephalitis): plotted T values of HIV without encephalitis (groups B,C) vs. HIV with encephalitis (group D) samples. Fourth color band (Impairment): plotted correlations of GCR scores, our measure of neurocognitive impairment, across all HIV subjects. Fifth color band (CPE score): plotted correlations of CPE scores across all HIV subjects. Red corresponds to genes with higher expression in HIV, impairment score (GCR), HIVE, and CPE score for color bands two through five, respectively. Note that the most significant correlations with disease tend to occur in FC. Arrows within top color band indicates that these modules are enriched for genes showing increased or decreased expression in hippocampus of Alzheimer’s disease (Blalock et al. 2004; p < 0.00002).

We applied WGCNA to probes detected in at least 6 samples, as reported by the Affymetrix cel files, including only a single probe for each gene. Hierarchical clustering led to the identification of several co-expression modules, ranging in number and size between the three regions. To assess the robustness of the co-expression module definition, we replicated module detection across all three brain regions. We find that of the 11, 13, and 14 modules in the FC, BG, and WM networks, respectively, six have correlates in all three networks and several more span two networks (Figure 3). Furthermore, when we use more sensitive module preservation statistics [17], we find that all of the common modules have strong preservation, except for the brown module (8), which has moderate preservation, and the red module (6), which is preserved well between FC and BG, but not as well in WM.

### Differential module eigengene expression across the four groups

Our module definition was solely based on the gene expression levels in brain tissue without consideration of HIV disease severity. In other words, modules were derived from all sample transcriptomes, regardless of group. To incorporate clinical and disease variables into the network analysis, WGCNA makes use of suitably defined gene significance measure. Here we defined the gene significance measure using ANOVA for testing differential module expression (as captured by the module eigengene) between the four groups. Thus, a large absolute value of the gene significance measure corresponds to a small 2-sided p-value. Note that we discuss only those modules that reached a non-adjusted significance threshold of p < 0.05, which represent trends in the data relating modules to clinical variables. Additional module information, including hub genes (i.e., correlations between individual genes and ME) and gene category enrichments (e.g., Swissprot, GO) for each module, is available in the Supplement Material [Additional file 2 Module and User Enrichment – All Modules.xls].

**Figure 4:**
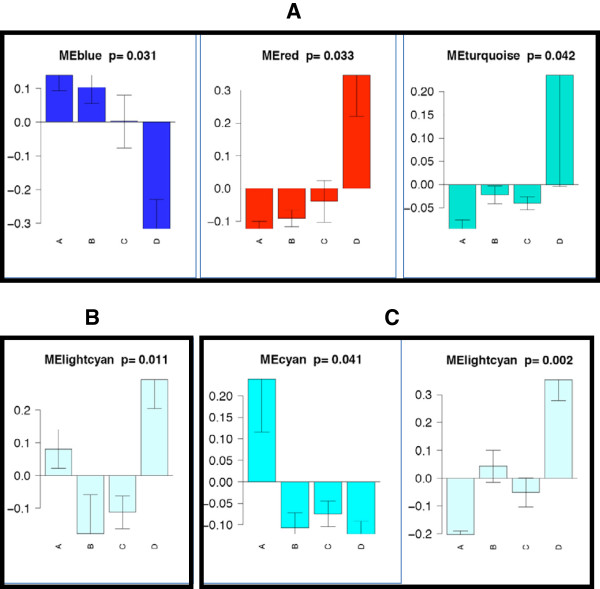
**Modules show different expression patterns across groups.** (**A**) The Blue, Red, and Turquoise modules from the frontal cortex show significant (Kruskal Wallis test p < 0.05) differences between groups. The Y axis corresponds to module eigengene expression. (**B**) The Light Cyan module shows increased expression with encephalitis in frontal white matter. In (**C**), the Cyan and Light Cyan modules show differential expression across groups in basal ganglia.

In the frontal cortex, the Red (Figure 3, Color band 6a), Blue (4a), and Turquoise (11a) modules showed trends towards differential expression across the four clinical groups (Figure 4, Panel A). The Red module (6a), here enriched with genes involved in transcription and DNA binding, as well as oligodendrocyte-related functioning, shows increased expression in the HIVE group (p = .033). The Blue module (4a) showed decreasing expression as disease severity increases (p = .031; Figure 4, Panel A). Top GO and Swissprot categories for the Blue module were hydrogen ion transporter activity, monovalent inorganic cation transporter activity, mitochondrion, and transporter activity. Finally, the Turquoise module (11a) also showed increased expression in the HIVE group (p = .042), and included the following GO and Swissprot categories: lysosome, lytic vacuole, glycosidase, antigen presentation, and interferon induction.

Within the white matter, the Light Cyan module (12c) showed a trend towards differential expression across the four clinical groups (p = .011; Figure 4, Panel B). The HIVE group demonstrated the highest expression level of this module. Interestingly, the HIV-seronegative group had higher expression than the other two HIV + groups. Top GO and KEGG pathways were hydrogen-transporting two-sector ATPase activity, carbohydrate metabolism, MHC-interacting protein, intracellular, and cell adhesion.

Finally, we examined modules in the basal ganglia network across all four clinical groups. The Light Cyan (Figure 3, Color ban 12b) showed between-group differences of p = 0.002, which was significant after correcting for multiple comparisons. The Cyan (15b) module showed a trend (p = 0.041). For the Light Cyan module, the HIVE group (Group D) had higher expression than the other two HIV + groups (Groups B & C), while the HIV-seronegative group (Group A) had the lowest expression (Figure 4, Panel C). Enrichment analysis of the Light Cyan module revealed the following top GO and Swissprot terms: immune response, response to biotic stimulus, defense response, and interferon induction. The top GO term for the Cyan module was transferase activity. The HIV-seronegative group had higher expression of the Cyan module than all three HIV + groups.

### Correlations between module eigengenes and clinical variables

Above we described differential expression analysis correlations between individual genes and the clinical variables (CPE or NCI). Here, we correlated module eigengenes with the clinical variables. The two approaches differ in that the differential expression analysis employs enrichment analysis as a method to interpret differentially expressed genes, whereas WGCNA identifies biological meaningful networks before examining their association with CPE and NCI. As such, WGCNA makes significantly fewer comparisons, thereby reducing the chance of Type I error. Here we report as trends those comparisons that reached a .05 threshold.

#### CPE

None of the modules were significantly correlated with CPE score in any of the brain regions.

#### NCI

**Figure 5:**
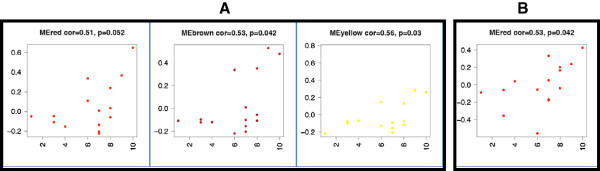
**Correlations between module eigengenes and GDS across all HIV + cases.** (**A**) In the frontal cortex, the Brown and Yellow modules have significant positive correlation with GDS, whereas the Red modules shows a mentionable trend. (**B**) In the frontal white matter, the red module has a significant positive correlation with GDS.

In the FC, the Brown (8a) and Yellow (5a) modules showed a trend towards correlation with NCI (Figure 5, Panel A). The Brown module was largely enriched with transcription genes, whereas the Yellow module was enriched for glycoprotein, signal and extracellular gene categories. Enrichment categories are listed in the Supplemental Material. The Red module (6a), largely enriched with transcription and DNA-binding genes, as well as oligodendrocyte functioning, also shows a notable trend (R = .51, *p* = .052). In all three instances, higher module expression correlated with greater NCI. Complete enrichment information for the FC and other brain regions is included as a Supplemental Table [Additional file 3 Module Enrichment NCI Only.xls].

In the WM, the Red module (6c), here enriched for oligodendrocyte marker genes, showed a trend towards correlation with NCI (R =0.53, p = 0.042; Figure 5, Panel B). Higher module expression correlated with greater NCI.

No significant correlations or trends between module expression and NCI among the HIV + cases were found in the BG.

### Study 2: comparison of HAND and Alzheimer’s disease transcriptomes

**Figure 6:**
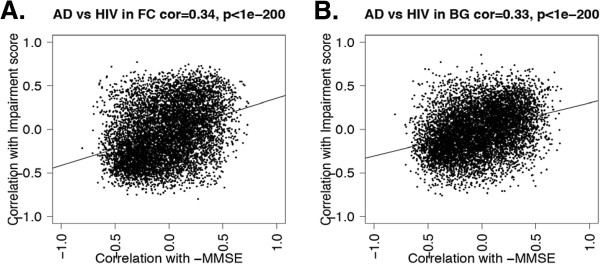
**Common impairment marker genes across regions and diseases.** Correlation between Impairment and gene expression in the hippocampus of Alzheimer’s disease is relatively consistent with correlation between impairment and gene expression in frontal cortex (**A**) and basal ganglia (**B**) of HIV-associated neurocognitive disorder. The x-axes represent correlation with GCR score in HIV brains, while the y-axes represent negative correlation with MMSE in the AD brains. Dots in the upper right of the plot represent genes whose increased expression in either HIV of AD would suggest impairment, while dots in the lower left represent genes whose decreased expression would suggest impairment.

In study 2 we sought to advance understanding of common mechanisms shared by HIV and AD by identifying genes that were similarly dysregulated in both diseases. A total of 234 genes were found to be upregulated and 413 genes downregulated in both diseases. As shown in Figure 6, the correlations between each of these genes and the respective neurocognitive scores indicate modest correlation between AD and HIV. Enrichment analysis revealed that genes in which expression decreased as NCI increased in both AD and HIV included those involved in cytoplasm, energy pathways, mitochondrion, tricarboxylic acid cycle, transit peptide, and synaptic vesicle, along with neuronal marker genes. Conversely, expression increased with NCI in genes involved in cell differentiation, activator, repeat, cell communication, regulation of transcription, and phosphorylation, along with marker genes for astrocytes (see Table 5).

**Table 5:** Enriched GO categories for common impairment genes

**Genes down-regulated with impairment in both AD and HIV**						
Gene Category	List Hits	List Total	Pop Hits	Pop Total	EASE score	Bonferroni
Cytoplasm	175	259	2277	4968	5.62E-13	1.40E-09
Energy pathways	26	259	128	5016	4.16E-09	1.04E-05
Mitochondrion	48	259	396	4968	3.27E-08	8.15E-05
Tricarboxylic acid cycle	10	259	20	5016	2.24E-07	5.58E-04
Transit peptide	28	217	184	4007	1.14E-06	2.85E-03
Synaptic vesicle	10	259	30	4968	1.31E-05	3.26E-02
**Genes up-regulated with impairment in both AD and HIV**						
Cell differentiation	10	131	90	5016	4.79E-13	1.40E-09
Activator	11	105	131	4007	1.89E-03	1.00E + 00
Repeat	42	105	1051	4007	1.98E-03	1.00E + 00
Cell communication	50	131	1363	5016	5.20E-03	1.00E + 00
Regulation of transcription	32	131	762	5016	5.70E-03	1.00E + 00
Phosphorylation	26	105	582	4007	6.18E-03	1.00E + 00

All genes in this list were correlated with MMSE in Blalock et al. [25] (R > 0.25), as well as with GCR in the current analysis of HIV brains (both FC and BG, R > 0.25).

Expression changes in modules identified via WGCNA of the HIV + sample are shown in Figure 3. This is strictly for illustrative purposes, as no formal analyses were conducted to assess degree of change. Modules with arrows are enriched for genes showing increased (↑) or decreased (↓) expression in the hippocampus of AD brains.

Furthering our understanding of which specific brain cells are involved in AD and HIV, results of cellular composition analysis are shown in Table 6. The analysis indicates that the impairment signature common to AD and HAND is likely marking neuronal loss/dysfunction accompanied by gliosis, or upregulation of astrocyte-mediated pathways.

**Table 6:** Brain cell-specific alterations common to AD and HIV

**Direction of change**	**Cell type enrichment**	**Bonferroni**
Down with impairment	Neuron (probable)*	3.68E-12
Down with impairment	Neuron (definite)*	1.13E-04
Up with impairment	Astrocyte (probable)	3.34E-12

* Definite (10+ fold) and probable (1.5+ fold) enrichment from Cahoy et al. [53].

Finally, we list genes that showed the strongest intramodule connectivity (>.70 or < −.70) in both AD and HIV in Table 7. These genes can be considered to have a central role in association with NCI in both AD and HIV. For the HIV cases we included only FC and BG as these regions are more similar to the hippocampus with regards to cell types. As shown in Table 7, among the upregulated genes there was a disproportionate number involved in cancer and tumor suppression. Downregulation of transcription occurred largely in genes involved in mitochondrial functioning and also genes implicated in several neurological diseases.

**Table 7:** Common genes correlated with neurocognitive impairment in AD and HIV

**Gene probe**	Function*	**Scaled intramodule connectivity kIN/max (kIN)**		
**Up with impairment**		**AD hippocampus**	**HIV frontal cortex**	**HIV basal ganglia**
CTDSP2	Cancer	1.0	1.0	1.0
SASH1	Cancer	0.84	0.95	--
FBXW12		0.86	--	0.79
HIPK2	Cancer	0.77	0.74	0.72
CASC3	Cancer	0.74	--	0.74
CEP350		0.72	0.90	--
PGF	Cancer	0.71	--	0.73
HS1BP3	Neurologic	0.71	--	0.72
**Down with Impairment**		**AD hippocampus**	**HIV frontal cortex**	**HIV basal ganglia**
SCG5	Neurologic	0.96	--	0.85
VDAC1	Mitochondria	0.95	0.99	0.92
KIAA1279	Neurologic	0.93	--	0.70
PFDN4		0.92	0.84	--
MDH1	Mitochondria	0.90	0.84	1.0
ATP5G3	Mitochondria	0.90	0.95	0.99
DYNC1I1	Neurologic	0.89	0.83	0.83
PCMT1	Neurologic	0.86	0.98	0.90
TOMM20	Mitochondria	0.86	--	0.92
KLC1	Microtubule	0.86	0.96	--
NDFIP1		0.86	0.73	0.84
KIFAP3	Neurologic	0.85	0.87	--
THYN1		0.85	0.74	0.78
GOT1	Mitochondria	0.84	0.83	0.84
TBC1D9		0.84	0.75	0.90
UCHL1	Neurologic	0.84	0.84	0.92
ACTR10		0.84	0.88	0.85
CISD1	Mitochondria	0.83	0.83	0.75
SMAP1		0.82	0.76	--
C14orf2		0.82	0.77	0.76
PEX11B		0.81	0.93	--
SLC4A1AP		0.81	0.71	--
COPS4		0.81	0.87	0.79
ITFG1		0.80	0.84	0.76
GLOD4		0.79	--	0.83
DNAJA2	Mitochondria	0.78	0.74	0.73
SUCLA2	Mitochondria	0.76	0.87	0.84
NDUFB6		0.76	--	0.78
EBNA1BP2		0.76	--	0.72
TUBA4A		0.75	--	--
HINT1	Cancer	0.75	0.86	0.80
ATP6V1E1	Mitochondria	0.74	--	0.75
GUCY1B3		0.72	0.83	0.91
ATP5H	Neurologic	0.72	0.73	--
GPI		0.70	0.75	--
BEX1	Neurologic	0.70	--	0.72

*Gene function was determined by reviewing information within the Online Mendelian Inheritance in Man database http://www.ncbi.nlm.nih.gov/sites/entrez?db=omim.

## Discussion

In the studies described within this paper, we employed WGCNA to further explore transcriptome changes within brain tissue of HIV + individuals. In addition, we directly compared transcriptome changes in brains obtained from individuals with HIV to those with Alzheimer’s disease. WGCNA has many advantages over traditional methods for differential expression analysis, including a focus on co-expression patterns thereby allowing for identification of biologically-relevant modules consisting of related genes, detection of hub genes that may eventually serve as targets for therapeutic modulation, and reducing data allowing for direct association analysis with disease-related variables [20-24,54].

HAND is a common consequence of HIV infection [55]. As suggested by the results of Gelman et al. [11], there may be two distinct etiologies for HAND. The first is associated with neuropathological findings of HIVE together with high brain viral load and global upregulation of inflammatory response genes and downregulation of neuronal pathways with the FC. The second lacks the classic neuropathological findings of HIVE and is associated with relatively low brain viral load. This type also has relatively little transcriptomic dysregulation, with the exception of upregulated endothelial cell type transcripts in the BG. Overall our results, largely indicated by statistical trends rather than significant findings, recapitulate the main findings of Gelman et al. [11]. For example, both the previous and current studies found significant upregulation of pathways involved in inflammation and neuroimmunity in those with HIVE. In the current study, this was reflected in the Light Cyan (12b) and Turquoise (11a) modules in the BG and FC, respectively, and to a lesser extent the Light Cyan module in the WM (12c). Note that our analysis differed from Gelman et al. in that we did not directly compare pairs of groups (e.g., A vs. D), but rather examined all groups via ANOVA. Further, Gelman et al. performed their enrichment analysis using Ingenuity software, and probed for changes in expression of more than two-fold. Conversely we utilized WGCNA, which allowed for the further analysis of these data and provided additional avenues of study based on this precious microarray resource. For instance, our module based analysis reveals that some of our modules relate to cell types. Specifically, we found that the Red module within the FC (6a), enriched with oligodendrocyte-related genes, was particularly elevated in the HIVE group (Group D), suggesting specific dysfunction of this cell type in those with HIVE. In addition, our module-based analysis led to somewhat different enrichment of GO categories, and also provides a framework for understanding how various pathways interact through co-expression. For example, Gelman et al. identified 2 upregulated canonical pathways within the FC of individuals with HIVE, consisting of interferon signaling and activation of IRF cytosolic pattern recognition receptors. In comparison, the WGCNA identified 2 upregulated modules, consisting of a much wider array of pathways. The first module indicated increased transcription, DNA binding, and oligodendrocyte-related functioning, while the second indicated increased lysosome, lytic vacuole, glycosidase, antigen presentation, and interferon induction. Further, while Gelman identified 11 downregulated pathways within the FC of those with HIVE, our analysis indicated a single module consisting largely of pathways involved in hydrogen ion transporter activity, monovalent inorganic cation transporter activity, mitochondrion functioning, and transporter activity. As such, it is possible that our single module represents a meta-pathway that encompasses many of the GO pathways identified by the previous approach, as well as some that were not detected previously. Together, the WGCNA reveals different canonical pathways associated with HIVE, allows for a more systems-based interpretation of the data, and provides insight into the interactive activity of the various canonical transcription pathways.

Further extending the findings of the previous study, we examined the relationship between HIV-associated neurocognitive impairment (NCI) and gene expression, without making a distinction between those with or without HIVE. The reason for this approach is based on the poor correlation between HIVE and neurocognitive deficits that are characteristic of HAND [2,3], suggesting that biological processes that do not inevitably culminate in HIVE underlie neurocognitive changes in those with HIV. We used two methods; 1) correlating standard differential gene expression with GDS, and 2) correlating modules derived via co-expression matrices with GDS. Our two methods revealed very different findings, both worthy of further exploration. First, using standard differential expression analyses coupled with a bioinformatics networking program, we identified three notable genes within the WM network. Expression of the inhibitor of DNA binding gene (ID2) was negatively correlated with NCI, such that expression of this gene decreased with higher NCI. The ID pathway has been implicated in reactive gliosis [56], and is upregulated by proinflammatory cytokines implicated in HAND [57]. Another apparently relevant gene showing modest connectivity is death-associated protein (DAP). The findings of Deiss [58] suggest that DAP is one of two genes that mediate gamma-interferon induced apoptosis. Importantly, gamma-interferon has long been known to play a significant role in HIV-related neuropathology [59,60]. Of note, the fact that ID2 and DAP were negatively correlated with NCI goes against expectation, considering their reported neurobiological roles and associations with HIV-related immune factors. A third relevant gene, that showed strong positive correlation with NCI, is GRK6. GRK6 is involved in dopamine sensitivity at the D2 receptor [61]. Dysfunction of the dopaminergic neural systems, such as the frontostriatal system, has been strongly implicated in the etiology of HAND [62-67]. As such, one possible scenario is that upregulation of GRK6 results in desensitization of D2 receptors within the frontostriatal system, leading to increased NCI. Desensitization of D2 receptors may in turn lead to upregulated transcription. The complex balance of dopaminergic tone, mediated largely by the D2 receptor, was recently described by Gelman et al., who found that downregulation of D2 transcripts in HIV + human prefrontal cortex was associated with more favorable neuropsychological and neuropathological outcomes [68], underscoring the possible role of this receptor in the etiology of HAND. Of note, chemokine (C-C motif) ligand 2 (CCL2), also known as monocyte chemotactic protein-1, is also shown in this network, and has been repeatedly implicated in HAND [69-72]. Secondly, we explored GO categories associated with NCI. Notably, statistically significant downregulated GO categories were found only in the FC. Categories included the mitochondrion, mitotic cell cycle, DNA repair, proteasome complex, and nucleus. Together, these findings implicate a general breakdown of cellular functioning within the FC, including energy depletion and catabolism of intracellular waste products. Dysregulation of proteasome functioning was implicated in the FC. Proteasome dysfunction was previously reported in studies that related gene expression to pre-mortem neurocognitive functioning [73,74]. Enriched GO categories were identified in all three brain regions, including transcription regulation in both FC and WM, and homo sapiens 19 in WM and BG. The homo sapiens 19 category contains a large number of brain-related genes. Finally, we employed WGCNA to examine co-expression modules associated with NCI. Largely confirming the GO analysis, we found modules in the FC largely representative of transcription and cellular signaling and glycoprotein functioning that showed significant positive correlation with impairment. However, in addition to these pathways, another module representative of oligodendrocyte functioning showed a notable trend (p = .052) for positive correlation in the FC. This module was also positively correlated with NCI in the WM. Within the Red module in WM (6c), hub genes included those implicated in other neurological conditions and CNS functioning, including WNK1 (linked to both cardiovascular [75] and Alzheimer’s disease [76]) and NEO1 (involved in nervous system development and apoptosis [77]). Interestingly, a larger module more prominently associated with oligodendrocytes (11c/Turquoise) does not show increased expression with NCI, suggesting that only a subset of oligodendrocyte-associated genes may be related to HAND. These results suggest that a more in depth look at oligodendrocytes in relation to HAND may be warranted, particularly given recent implications of the role of oligodendrocytes in HAND and HIVE [78-80], as well as other neurodegenerative diseases, including AD [81,82]. Indeed, considering the prominence of myelin pallor and gliosis as early neuropathological findings in AIDS patients, the role of oligodendrocyte-associated gene dysgregulation is not unexpected. Importantly, as these genes did not individually show significant correlations with NCI, they would not have been detected in a standard differential expression analysis.

Both methods (standard differential expression analysis and WGCNA) here provide complementary interpretation of changes that occur in the brain with NCI, and together provide a more complete look at NCI than either method alone. For example, both methods suggest that NCI is the result of increased transcription activity within the FC and WM. Further, both implicate decreased mitochondrial activity in the FC, and in the case of WGCNA, frontal white matter as well. Beyond that, standard analysis suggests that NCI is associated with downregulation of various networks or pathways involved in cellular functioning, energy metabolism, and proteasome functioning within the FC, as well as upregulation of categories such as transcription regulation in FC and WM, and Homo Sapiens 19 genes in WM and BG. Further, specific genes implicated include those involved in dopamine receptor sensitivity, apoptosis, and reactive gliosis. Conversely, WGNCA suggests that NCI is associated with increased transcription within the FC and WM. However, it also implicates oligodendrocyte dysfunction. Notably, the WGCNA did not identify any modules within the BG that were associated with NCI. Such divergent results are important, as they provide alternative directions for future investigations.

In study 1 we also examined the relationship between transcriptomic changes and antiretroviral therapy regimen, or more specifically CPE. The CPE is based on pharmacokinetic and pharmacodynamic characteristics of antiretroviral medications, including their ability to cross the blood brain barrier and eradicate HIV within the CNS [29]. In the current study, using differential expression analysis, only a few genes were found to have modest correlations with CPE, and no GO categories were identified. Follow-up analysis with GNCPro in the WM revealed only a single gene with notable connectivity; TNPO3. TNPO3 is required for HIV-1 integration into the host DNA, and higher CPE would be expected to decrease its expression [51,52]. Counter intuitively, expression of this gene was positively correlated with CPE in the WM. Even when using WGCNA, no co-expression modules were found to be associated with CPE, suggesting that higher penetrating regimens do not have a significant impact upon gene expression. While ours is the first study to examine the association between CPE and brain transcriptome in HIV, the relationship between combination antiretroviral therapy (cART) use and brain gene expression was recently described by Borjabad et al. [83]. Notably, they found that cART-treated cases had transcriptome signatures that more closely resembled those of HIV-seronegative cases. Further, brains of individuals who were taking cART at the time of death had 83-93% fewer dysregulated genes compared to untreated individuals. Despite this, in both treated and untreated HIV + brains there were approximately 100 dysregulated genes related to immune functioning, interferon response, cell cycle, and myelin pathways. Perhaps helpful in explaining our findings, gene expression in the HIV + brains did not correlate with brain viral burden, suggesting that even high CPE regimens, which have been shown to reduce CSF viral load [84], may not reduce transcriptomic dysregulation. Indeed, the absence of an association between CPE and brain transcriptome would help to explain the overall equivocal results thus far of studies examining the relationship between CPE and HIV-related NCI [31-33,85].

Study 2 sought to identify common transcriptome changes in HIV and AD that were related to NCI. Findings indicate some overlapping biological pathways underlying these two diseases. In total, 234 identical genes were upregulated and 413 downregulated in both diseases. Further, there was a modest correlation between HAND and AD in the level of correlation with cognitive impairment. More specifically, genes strongly correlated with NCI in HAND tend also to be strongly correlated with NCI in AD. Suppressed biological pathways associated with NCI in both diseases include cytoplasm, energy pathways, mitochondrion, tricarboxylic acid cycle, transit peptide, and synaptic vesicle. Conversely, expression of genes involved in cell differentiation, activator, repeat, cell communication, regulation of transcription, and phosphorylation increased with severity of NCI in both diseases. With regards to specific cell types, expression of neuronal marker genes was reduced with increasing NCI in both diseases, whereas expression of marker genes for astrocytes increased with NCI. Together, a picture emerges of waning neuronal functioning and waxing astrocytosis underlying the progressive neurocognitive decline in both diseases. This is further indicated by the results of cellular composition analysis, which found that neuronal loss/dysfunction along with gliosis underlies the impairment signature common to HIV and AD, a finding that is consistent with previous studies of AD [86,87] and HAND [88-90]. Notably, examination of genes with greatest intramodular connectivity that are common between AD and HIV confirmed the down-regulation of mitochondrial-related genes in relation to NCI in both diseases; however, it also revealed a disproportionate number of cancer-related genes that were upregulated in both groups. This included genes in which upregulation is implicated in cancer (CTDSP2, CASC3, PGF) and those that are thought to be involved in tumor suppression (SASH1, HIPK2). The significance of these findings is unclear. Note that a recently published meta-analysis compared brain transcriptomes (frontal grey and/or frontal white matter) from individuals with HIVE and/or HAND to those from individuals with AD (various anatomic locations), without consideration of NCI [91]. Due to the different methodologies used, and the lack of specific anatomical focus (which as demonstrated here can lead to vastly different results), these two studies are not directly comparable.

While the findings of the current studies are compelling, they should be considered with the following caveats. First, the HIV + sample, while well-characterized both pre and post-mortem, was small. This in turn limited the power of our statistical analyses, and as such we have largely reported trends rather than statistically significant findings. While this limits the impact of our findings, we hope that they may still stimulate questions and new avenues for investigation. Second, the association of pre-mortem clinical data with post-mortem transcriptome data is fraught with difficulties, including variable time ranges between data collection, questionable reliability of psychometric testing among the very ill, uncertainty of adherence to ARV regimen, and effects of different causes of mortality upon brain transcription, to name a few. Finally, the comparison of AD and HIV brains involved different anatomical regions, which may have distinct transcription pathways regardless of disease state.

## Conclusion

In summary, we provide here a systems biological interpretation of brain transcriptome data derived from HIV + individuals and those with Alzheimer’s disease. Our results build upon previous findings that utilized a different grouping and analytic approach, and add an important dimension to understanding HIV-neuropathogenesis from a systems perspective. In addition, the identification of common pathways and genes associated with NCI in HIV and Alzheimer’s disease is a novel finding that we believe warrants further investigation.

## Competing interests

Dr. Horvath developed weighted gene coexpression network analysis, the primary analytic method described in this paper. He does not make direct profits from this tool, which is freely available online.

## Authors’ contributions

AL, JM, and SH initially conceived the projects and developed the methods and statistical approach. AL and JM were the primary authors of the text. JM and SH conducted the various statistical analyses and were also involved in the interpretation of the results. BG conducted the initial microarray of HIV + brains, and provided the data. He was also involved in manuscript preparation and provided invaluable input. PS conducted the GNCPro analysis and provided related interpretation. IG, SM, ES, DC, and CH are all key members of the NNTC, and were instrumental in providing data for the study, interpretation of results, and manuscript preparation. All authors have approved of the final version of this manuscript.

## Pre-publication history

The pre-publication history for this paper can be accessed here:

http://www.biomedcentral.com/1755-8794/6/4/prepub

## Supplementary Material

Additional file 1 Correlations between gene expression and clinical variables (CPE scores and neurocognitive impairment). Click here for file

Additional file 2 Hub genes and gene ontology enrichment for all modules found among the four clinically/neuropathologically-defined groups. Click here for file

Additional file 3 Gene ontology enrichment for the modules related to neurocognitive impairment in the frontal cortex. Click here for file
